# Nutrient intake patterns and breast cancer risk among Jordanian women: a case-control study

**DOI:** 10.4178/epih.e2019010

**Published:** 2019-03-30

**Authors:** Reema Fayez Tayyem, Reema Ibrahim Mahmoud, Muna Hussien Shareef, Lina Salah Marei

**Affiliations:** 1 Department of Nutrition and Food Technology, Faculty of Agriculture, University of Jordan, Amman, Jordan; 2 Al-Basheer Hospital, Ministry of Health, Amman, Jordan; 3 King Hussein Cancer Center, Amman, Jordan

**Keywords:** Breast cancer, Diet, Nutrient pattern, Case control studies, Jordan

## Abstract

**OBJECTIVES:**

Breast cancer (BC) is the most common type of cancer worldwide. Globally, BC is rapidly becoming a major common health problem among women. This study aimed to evaluate the association between nutrient intake patterns and BC risk among Jordanian women.

**METHODS:**

A total of 400 Jordanian women 20-65 years of age were recruited in this case-control study. Two hundred women recently diagnosed with BC were matched in age, income, and marital status to 200 BC-free women. A food frequency questionnaire was used to assess nutrient intake patterns.

**RESULTS:**

In this study, 3 nutrient intake patterns were identified: a high vitamin C and β-carotene nutrient intake pattern; a high calcium, phosphorus, and vitamin D nutrient intake pattern; and a high-fat nutrient intake pattern. A significant increase in BC risk was associated with the high vitamin C and β-carotene nutrient pattern (the highest for the fourth quartile; odds ratio [OR], 5.42; 95% confidence interval [CI], 2.11 to 13.91; p_trend_=0.001). In the high calcium, phosphorus, and vitamin D nutrient pattern, a significant inverse trend was detected for the risk of BC. The high-fat nutrient pattern showed a significant direct association with BC risk in the third (OR, 3.88; 95% CI, 1.58 to 9.51) and fourth (OR, 3.87; 95% CI, 1.53 to 9.77) quartiles (p_trend_=0.001).

**CONCLUSIONS:**

A significant increase in BC risk was detected for the high vitamin C and β-carotene nutrient intake pattern and the high-fat nutrient intake pattern. However, for the high calcium, phosphorus, and vitamin D nutrient intake pattern, a significant inverse trend was observed.

## INTRODUCTION

Cancer is a leading cause of death in both more and less economically developed countries. The burden of all types of cancer is expected to grow worldwide due to population growth and aging [1]. Approximately 18.1 million new cancer cases and 9.6 million deaths occurred in 2018 worldwide [2]. Breast cancer (BC) is the most common type of cancer; it affects women throughout the world, and is among the top 5 cancers in Jordan [3]. In 2014, there were 1,187 newly diagnosed cases of BC in both genders, accounting for 20.8% of all newly diagnosed cancer cases [3].

Although a number of risk factors have been identified for BC, some are difficult to modify [4]. While dietary and nutrient intake patterns, physical activity (PA), alcohol drinking, body mass index (BMI), and smoking are modifiable risk factors of BC, genetic factors, age, and gender are considered to be non-modifiable risk factors [1,4,5]. There is accumulating evidence associating combined nutrient and dietary patterns, single micronutrients, macronutrients, and food items with the pathogenesis of BC [4,6,7]. However, most of these relationships remain controversial [4,6,7]. Although limited evidence is currently available in support of probable causal judgments regarding the etiological role of specific dietary factors [8], diet nonetheless plays a role in the continuous increase of BC incidence [9]. The associations of dietary and nutrient intake patterns with BC have been investigated in numerous studies [1,9-11]. The findings of these studies were inconclusive, and most studies have examined the risks associated with specific foods and nutrients, rather than measures of overall dietary patterns [1, 9-11]. Therefore, the objective of this study was to investigate associations between nutrient intake patterns and BC risk among Jordanian women.

## MATERIALS AND METHODS

The study was conducted in accordance with the ethical standards of the responsible committee on human experimentation and with the Helsinki Declaration of 1975 and subsequent amendments. The proposal was approved by the Institutional Review Board of the 2 participating hospitals: King Hussein Cancer Center (KHCC) and Al-Basheer (16 KHCC 57 and 2,250, respectively).

### Study design

A case-control design was used to evaluate the nutrient intake patterns a risk factor associated with BC among Jordanian women.

### Sample enrollment

Two hundred patients who had recently been diagnosed (up to 3 months from diagnosis) with BC were enrolled in the study through convenience sampling. All BC patients were taken from KHCC and Al-Basheer Hospitals, the main 2 hospitals in Jordan that offer cancer therapy. Data was collected between October 2016 and September 2017. The control group was included from the community (the employees and visitors of KHCC and Al-Basheer Hospitals, as well as patients’ accompanying persons, with the exclusion of first-degree relatives) who underwent either a mammogram or clinical examination to verify that they were free of BC. The ratio of cases to controls was 1:1. Matching between the patients and control subjects was performed for the following variables: age, income category, and marital status. The inclusion criteria for the patients were: a recent diagnosis of BC (up to 3 months from diagnosis), Jordanian nationality, age between 20 years and 65 years, and the ability to communicate verbally. The exclusion criteria included critical or terminal illness, hospitalization, an inability to communicate verbally, and suffering from other types of cancer or other diseases that required a specific diet. Data from patients who decided to terminate the interview, gave unrealistic answers during the interview, or were diagnosed at an age older than 65 years were excluded (16 cases and 24 controls). The sample size was calculated as: number (women diagnosed with BC in 2014)=1,174; confidence interval (CI), 95%; margin of error, 5%; and response distribution, 85%. The calculated sample size was 170. In general, approximately 20% of the population of patients diagnosed with BC in 2014 in Jordan was enrolled.

### Setting

For the cases, the hospital setting was utilized for data collection. Hospitals with specialized oncology units were included (KHCC and Al-Basheer Hospitals). The outpatient department in each hospital was the setting for data collection. Permission from each hospital to use a private room in a suitable physical condition to carry out the interviews was obtained. For the controls, KHCC and Al-Basheer Hospitals were the setting for data collection.

### Data collection

A 3-part package was used to collect data for the purposes of the study. The package consisted of 3 structured questionnaires: a personal information sheet, a food frequency questionnaire (FFQ) [12], and 7-day physical activity recall (PAR) [13]. A face-to-face interview was used for data collection and the questionnaire was completed by a trained researcher.

#### Personal information sheet

This sheet was composed of questions related to age, marital status, education, employment, monthly family income, residency area and house condition, smoking status, medication, and previous and current health problems.

#### Dietary assessment

Information on diet was collected using the validated Arabiclanguage FFQ [12]. The FFQ included 109 questions on food and beverages. Response categories provided in the FFQ were <1/mo, 2-3/mo, 1-2/wk, 3-4/wk, 5-6/wk, 1/d, 2-3/d, 4-5/d, and 6/d. For some other food items, the response categories provided in the FFQ were never, 25% of the time, 50% of the time, 75% of the time, and all the time. Standardized food models (Nasco, Fort Atkinso, WI, USA) and standard measuring tools were used to help participants to estimate the consumed portion size. The food lists in the modified FFQ questions were arranged based on types of foods: 21 items for vegetables; 16 items for meat, such as red meat (lamb and beef), chicken, fish, processed meats, and others; 21 items for fruits and juices; 9 items for milk and dairy products; 8 items for cereals; 4 items for beans; 4 items for soups and sauces; 5 items for drinks; 9 items for snacks and sweets; and 14 items for herbs and spices. After completing the FFQ, the selected frequency category was converted to a weekly intake. To quantify participants’ nutrient intake, software (ESHA Food Processor SQL version 10.1.1; ESHA, Salem, OR, USA) that calculates vitamin and mineral intake based on food intake was used.

#### Physical activity levels

The physical activity levels (PARs) questionnaire, developed by Sallis et al. [13] for the Stanford Five-City Project, was used to determine the level of PA among participants on a weekly basis. When using the PAR, it is necessary to consider the frequency, intensity, time, and type of the PA. The 7-day PAR is a structured interview that assesses participants’ recall of time spent engaging in PA over a 7-day period. It covers different levels of PA, such as aerobic exercise, work-related activities, gardening, walking, recreation, and leisure-time activities [13]. We measured PA using a metabolic equivalent (MET) score. An estimate of total METs was calculated.

### Anthropometric measurements

Measurements of weight were taken to the nearest 0.1 kg, with minimal clothing and without shoes, using a calibrated portable scale. Height was measured to the nearest 0.5 cm with participants in the full standing position without shoes using a calibrated portable measuring rod. BMI was calculated as the ratio of weight in kilograms to the square of height in meters. Height and weight were measured according to Lee & Nieman [14].

### Human protection

Representatives from each hospital called each participant to invite them to participate in the research. During the first meeting with the participants, the investigator explained the purpose of the study and allowed participants to read the consent form before signing it. Each participant understood that she could withdraw from the study at any time she wanted and that doing so would not harm her in any way. The potential risks and potential benefits were also explained in detail, and it was communicated that the present study posed no risk. A signed consent was obtained before data collection. The information obtained from patients was treated confidentially. Only oncologist knew the patients’ names, and she was the only one who gave them their ID number. All used tools and instruments (questionnaires) were labeled with patient ID.

### Statistical analysis

Descriptive analyses were conducted to examine the frequency of different variables. The chi-square test was used to detect significant differences among categorical variables. The t-test was used to assess differences between continuous variables from the cases and controls, which are presented as mean±standard deviation (SD). Logistic regression was used to calculate odds ratios (ORs) and 95% CIs, while linear regression was used to calculate p_trend_ values. Age (continuous), BMI (continuous), PA level (continuous), total energy intake (continuous), occupation, education level, marital status, and family history for the BC participants were evaluated as potential confounders [14]. The significance level was set at p<0.05. All statistical analyses were conducted in SPSS version 22.0 (IBM Corp., Armonk, NY, USA).

Principal component analysis of the entire population was conducted using a selected set of 28 major nutrients, macronutrients, and micronutrients to derive dietary patterns (Table 1). Factors were retained based on an eigenvalue of >1.0 for the scree plot and factor interpretability. Varimax rotation was then applied to the factor-loading matrix to review the correlations between variables and factors. Nutrients with absolute rotated factor loadings >0.4 were considered to be significant contributors to the patterns, and those with an absolute rotated factor loading greater >0.6 on a given factor were used to name the factors. Cases and controls received an individual factor score for each identified pattern to indicate the degree to which each subject’s diet conformed to that pattern [14].

## RESULTS

### Characteristics of the study sample and lifestyle risk factors

The results of this case-control study highlighted the associations of some dietary and lifestyle risk factors with the risk of developing BC. Table 1 shows participants’ age, anthropometric measurements, and socio-demographic and health characteristics. The average age of cases and controls was 48.9±0.6 years and 47.5±0.6 years, respectively. A significant difference was found between cases and controls for BMI. Controls showed a significantly higher level of PA (3,464.7±1,205.6 METs) than cases (1,425.4±133.3 METs). Other variables that differed significantly been cases and controls included the number of pregnancies, smoking, education level, employment status, and family history of BC.

### Factor loading matrix for the nutrient intake patterns identified in the study sample

Table 2 shows the factor-loading matrix for the 3 retained factors. These factors explained 48.5% of the total variance in the original dataset. Rotation had the effect of making loadings either all positive or all negative for each factor, in such a way that only the magnitude of each loading (and not its sign) was used to name the factors. The first factor (a high vitamin C and β-carotene nutrient intake pattern) had the greatest loadings for animal fats, cholesterol, and saturated fatty acids in addition to fiber, folic acid, vitamin C, and iron. The second factor (a high calcium, phosphorus, and vitamin D nutrient intake pattern) had the greatest absolute loadings for calcium, cholesterol, saturated fatty acids, riboflavin, zinc, vitamin D, and phosphorus. The third factor (a high-fat nutrient intake pattern) was characterized by the greatest loadings for vegetable fat, vitamin E, monounsaturated fatty acids (MUFAs), polyunsaturated fatty acids (PUFAs), and vegetable protein.

### Associations of the identified nutrient intake patterns and breast cancer risk

The ORs and corresponding 95% CIs of the BC cases and controls by quartiles of factor scores and continuous factor scores for the 3 nutrient intake patterns are shown in Table 3. The results refer to the composite model including all 3 nutrient intake patterns simultaneously, together with the relevant confounding variables. A significant association was found for all 3 factors and BC. A significantly higher risk of BC was associated with the high vitamin C, β-carotene nutrient pattern (the highest in fourth quartile: OR, 5.42; 95% CI, 2.11 to 13.91; p_trend_=0.001). For the high calcium, phosphorus, and vitamin D nutrient pattern, a significant inverse (i.e., protective) trend was detected against BC. The high-fat nutrient pattern showed a significant association with BC risk in the third (OR, 3.88; 95% CI, 1.58 to 9.51) and fourth (OR, 3.87; 95% CI, 1.53 to 9.77) quartiles, with a p_trend_ of 0.001.

## DISCUSSION

The results of this study add to the evidence for an association between BC risk and diet and lifestyle behaviors. In our study, a significant difference between cases and controls in BMI was detected; furthermore, the risk of BC increased as the BMI increased from normal body weight to overweight or obesity. BMI has been found to be associated with the risk of BC incidence, and this association has been reported in many studies [8,15-17]. Additionally, the present study revealed an inverse association between PA and BC. This is in agreement with several studies indicating that PA was significantly associated with a reduced risk of BC [18-21]. PA might reduce BC risk through several biological mechanisms, including its impact on adiposity, modulation of sex hormones, and reduction in insulin resistance, adipokines, and inflammatory markers [18-21].

Regarding diet, 3 nutrient intake patterns were identified in this study: a high vitamin C and β-carotene nutrient pattern, which was associated with an increased risk of BC; a high calcium, phosphorus, and vitamin D nutrient pattern, which showed a protective effect against BC; and a high-fat nutrient pattern, which was also associated with a higher risk of BC.

Although several studies demonstrated that vitamin C and β-carotene may favorably influence BC risk because of their antioxidant properties [22-25], our study showed that high intake of those 2 nutrients may be associated with higher risk of developing BC among Jordanian women. However, there is somewhat weaker evidence for a link between vitamin C and β-carotene with hormone-sensitive BC [24]. A plausible explanation for the association between antioxidants and BC risk was suggested by Salganik [26]. Those authors reported that reactive oxygen species in moderate concentrations act as indispensable mediators of cancer-protective apoptosis and phagocytosis, and that in people with a low level of reactive oxygen species, an excess of antioxidants can block these cancer-preventive mechanisms and promote cancer [26]. An excess of antioxidants, which interferes with apoptosis, can be cancer-promoting in people who are regularly exposed to the effect of environmental carcinogenic factors (tobacco smoke, industrial pollutants) that result in a high accumulation of pre-cancerous and cancerous cells [26]. Nagel et al. [27] demonstrated no associations between BC risk and dietary vitamin C and β-carotene. In contrast, Bakker et al. [24] documented that higher concentrations of plasma vitamin C and β-carotene reduced the risk of BC.

The second nutrient pattern in our findings was characterized by high calcium, phosphorus, and vitamin D intake, which may reflect high levels of dairy product consumption. According to our results, this nutrient pattern was significantly protective against BC. This finding is in agreement with the previous findings of Knekt et al. [28], who demonstrated that milk consumption exerted a protective effect against the risk of BC. Furthermore, milk comprises a complex mixture of macronutrients and micronutrients. The observed association may be caused by the protective effect of some or a combination of these nutrients [28]. This is also similar to results of Dong et al. [29], who documented that increased consumption of total dairy food, but not milk, may be associated with a reduced risk of BC. Other studies found that dairy consumption was not strongly related to BC risk [30,31]. Zhang et al. [31] supported the hypothesis that a high intake of dietary calcium may exert a protective effect on BC risk.

In the third nutrient pattern, which was characterized by high fat intake, a higher intake of MUFAs and PUFAs was found to significantly increase the risk of BC. In particular, among MUFAs, the *trans* form is likely to have been responsible for this relationship. The association between BC risk and *trans* fatty acids in general and MUFAs in the *trans* form was precisely studied by Chajès et al. [32]. Those authors found that intake of the *cis* forms of MUFAs was unrelated to BC risk, while a high serum level of *trans* MUFAs, presumably reflecting a high intake of fried foods, likely contributed to an increased risk of invasive BC in women [32]. A finding in this study (not presented in the Results section) was that *trans* fat intake in cases was higher than in controls and the percentage of total *trans* fats among total nutrients intake showed a significant trend (p<0.05) for BC risk. Furthermore, Wirfält et al. [33] concluded that PUFA intake increased the risk of BC. PUFAs produce eicosanoids, which increase the risk of BC. Besides, PUFAs have many double bonds that are easily oxidized, enhancing cellular damage [33]. Similarly, Chajès et al. [32] documented that PUFA intake significantly increased the risk of BC. However, Sczaniecka et al. [34] found that total PUFA intake was not associated with BC. Farvid et al. [35] reported the same pattern in their study. They found that a higher intake of fats was associated with a higher risk of BC; in addition, intake of saturated fatty acids (SFAs) and MUFAs was also related to an increased risk of BC among all participants. This was in agreement with the results of Salarabadi et al. [15], who suggested that SFA intake increases the risk of BC. In contrast, the findings of Alexander et al. [36] were not supportive of a positive independent association between consumption of SFA and BC.

In a study of this type, we rely greatly on the ability and memory recall of participants to accurately and carefully provide information from a period when it was not necessarily important to remember the details of meals that were consumed, or PAs that were undertaken. It is understandable that some individuals may have a better recall than others, and that biases may exist in the minds of those being interviewed, and indeed, in the interviewer. We also did not take into account the possible effects of cooking on the bioavailability of the various nutrients, although we attempted to control for a range of potential confounders. Alcohol consumption was not measured because its consumption is culturally discouraged in Jordan. Only 2 women reported alcohol consumption. Although the sample size was small, it was representative, as confirmed by statistical calculations. The enrollment of the participants utilized convenience sampling, and was based on their agreement to participate in the study, which imparts some degree of bias in terms of sample selection. Jordan is a small country with an estimated population in 2012 of approximately 9.5 million people, and a cancer diagnosis rate for BC of 32.1 per 100,000. In 2014, a total of 1,187 newly diagnosed BC cases in both genders were reported by the Jordanian Cancer Registry. We had difficulties in recruiting more participants to increase our sample size.

A major strength of our study is the use of a validated and detailed FFQ to collect dietary data from our study population. Even though dietary data were collected only once, the FFQ has been reported to be an adequate instrument for measuring macronutrient and micronutrient intake. However, confirmative studies should verify and extend the presented data on Jordanian dietary habits in order to establish recommendations for people in Jordan to reduce BC incidence.

In conclusion, 3 nutrient intake patterns were identified in this study: a high vitamin C and β-carotene nutrient intake pattern, which was associated higher BC risk; a high calcium, phosphorus, and vitamin D nutrient intake pattern, which showed a protective effect against BC; and a high-fat nutrient intake pattern, which was also associated with a higher risk of BC.

## Figures and Tables

**Table 1.** Characteristics of the study participants

Characteristics	Cases (n=200)	Controls (n=200)	p-value
Age (yr)	48.9 ± 0.6	47.5±0.6	0.106
Height (cm)	159.3±0.4	161.7±0.4	0.001
Weight (kg)	75.7±1.0	73.2±0.9	0.080
BMI (kg/m²)	29.8±0.4	27.9±0.4	0.001
No. of pregnancies	4.6±0.2	3.4±0.2	0.001
No. of miscarriages	1.1±0.1	0.90±0.1	0.226
Duration of lactation (mo)	8.2±0.5	7.7±0.5	0.599
Physical activity (METs)	1,425.4±133.3	3,464.7±1,205.6	0.019
Marital status			0.789
Married	154 (77.0)	156 (78.0)	
Single	26 (13.0)	29 (14.5)	
Divorce	8 (4.0)	5 (2.5)	
Widow	12 (6.0)	10 (5.0)	
Education level			0.001
Illiterate	12 (6.0)	5 (2.5)	
School	52 (26.0)	12 (6.0)	
High school	65 (32.5)	46 (23.0)	
Diploma	47 (23.5)	65 (32.5)	
Bachelor	14 (7.0)	55 (27.5)	
Master’s degree	7 (3.5)	12 (6.0)	
Doctorate degree	3 (1.5)	5 (2.5)	
Work status			0.001
Yes	53 (26.5)	89 (44.5)	
No	147 (73.5)	111 (55.5)	
BMI categories			0.013
Underweight	0 (0.0)	1 (0.5)	
Normal weight	33 (16.5)	58 (29.0)	
Overweight	91 (45.5)	70 (35.0)	
Obese	76 (38.0)	71 (35.0)	
Smoking			0.001
Yes	40 (20.0)	14 (7.0)	
No	160 (80.0)	186 (93.0)	
Family members diagnosed with cancer			0.001
Yes	109 (54.5)	73 (36.5)	
No	91 (45.5)	127 (63.5)	

Values are presented mean±standard diviation or number (%). BMI, body mass index; MET, metabolic equivalent.

**Table 2.** Factor loading matrix for the 3 major dietary patterns identified in the study sample^1^

Components	Dietary patterns		
	High-vitamin C and β-carotene	High-calcium, phosphorus, and vitamin D	High-fat
Energy (kcal)			0.244
Vegetable protein	0.213		0.606^2^
Animal protein	-0.206	0.438	
Vegetable oils			0.302
Animal fats	-0.311		0.334
Cholesterol	-0.332	0.416	
Saturated fat	-0.306	0.671^2^	
Monounsaturated fat			0.851^2^
Polyunsaturated fat			0.634^2^
Carbohydrates	0.439	-0.440	-0.372
Starch	0.453		
Sodium			
Calcium		0.886^2^	
Potassium	0.835^2^	0.348	
Phosphorus	0.211	0.865^2^	
Iron	0.480	-0.283	-0.370
Zinc			
Vitamin B_1_			-0.715^2^
Vitamin B_2_	-0.263	0.448	-0.399
Vitamin C	0.642^2^		
Vitamin B_6_	0.358		
Folate	0.421		-0.528
Niacin			-0.484
Retinol	-0.416	0.441	
Beta-carotene	0.742^2^		
Vitamin D		0.838^2^	
Vitamin E	0.344		0.719^2^
Variance explained (%)	4.39	4.04	3.97

1 Absolute values < 0.20 were excluded from the table for simplicity.

2 Absolute values ≥ 0.6.

**Table 3.** Associations of the identified nutrient intake patterns and breast cancer risk^1^

Dietary pattern	Q1	Q2	Q3	Q4	p_trend_
High vitamin C and β-carotene nutrient pattern					
Case (n)	18	24	25	42	0.001
Control (n)	47	41	39	23	
OR (95% CI)	1.00 (reference)	1.57 (0.61, 4.05)	2.24 (0.87, 5.77)	5.42 (2.11, 13.91)	
High calcium, phosphorus, and vitamin D nutrient pattern					
Case (n)	29	30	20	30	0.001
Control (n)	35	35	46	34	
OR (95% CI)	1.00 (reference)	0.76 (0.31, 1.85)	0.47 (0.19, 1.12)	0.67 (0.28, 1.64)	
High-fat nutrient pattern					
Case (n)	19	16	37	37	0.001
Control (n)	46	49	28	27	
OR (95% CI)	1.00 (reference)	0.77 (0.29, 2.01)	3.88 (1.58, 9.51)	3.87 (1.53, 9.77)	

OR, odds ratio; CI, confidence interval.

1 Adjusted for age, martial status, education, work, income, physical activity, smoking, family history, health problem, number of pregnancies, lactation, contraceptives, hormonal replacement therapy.
